# In Vitro Evaluation of Three *Pisum sativum* L. Varieties to Partially Replace Soybean and Corn Meal in Dairy Cow Diet

**DOI:** 10.3390/ani15060855

**Published:** 2025-03-17

**Authors:** Maria Ferrara, Emanuele D’Anza, Teresa Montefusco, Piera Iommelli, Barbara Piccirillo, Alessio Ruggiero, Alessandro Vastolo

**Affiliations:** Dipartimento di Medicina Veterinaria e Produzioni Animali, University of Napoli Federico II, 80137 Napoli, Italy; maferrar@unina.it (M.F.); emanuele.danza@unina.it (E.D.); teresa.montefusco@unina.it (T.M.); piera.iommelli@unina.it (P.I.); barbara.piccirillo@unina.it (B.P.); alessandro.vastolo@unina.it (A.V.)

**Keywords:** energy, protein, starch, soybean meal, volatile fatty acids, ammonia

## Abstract

Pea (*Pisum sativum* L.) seeds are valuable feed ingredients due to their high-quality protein and starch digestibility, making them a viable alternative to soybean meal and corn grain. This study evaluated the nutritional value of three commercial pea varieties (Ganster, Peps, and Poseidon) through in vitro trials. Each variety was incorporated into an experimental diet (GNS, PES, and PNS) for dairy cows, partially replacing soybean and corn meals. These diets were compared to a control diet containing only soybean and corn meals. All diets were incubated anaerobically for 120 h with dairy cow rumen liquor. Results showed that GNS and PES diets enhanced protein degradability (*p* < 0.05) and fermentation kinetics (*p* < 0.001). Additionally, all experimental diets reduced ammonia production (*p* < 0.001), while the PES diet increased (*p* < 0.001) volatile fatty acid production. Among the tested varieties, Peps demonstrated the greatest potential by improving protein metabolism and volatile fatty acid production. These findings suggest that pea grains can be a suitable alternative in dairy cow diets, supporting efficient ruminal fermentation and nutrient utilization.

## Simple Summary

Pea (*Pisum sativum* L.) seeds are a valuable feed ingredient due to their high protein content and starch digestibility, making them a promising alternative to soybean meal and corn grain in dairy cow diets. This study evaluated three commercial pea varieties (Ganster, Peps, and Poseidon) by incorporating them into experimental diets GNS (Ganster), PES (Peps), and PNS (Poseidon) to partially replace soybean meal and corn meal. These diets were compared to a standard control diet (CTR) through in vitro trials using dairy rumen liquor incubated for 120 h. Results indicated that pea-based diets maintained organic matter digestibility and gas production while improving protein degradability and fermentation kinetics. Furthermore, all experimental diets reduced ammonia production, which can contribute to improved nitrogen efficiency and decreased environmental impact. The PES diet increased volatile fatty acid production, supporting better energy utilization by cows. Among the tested varieties, Peps showed the most promising results by enhancing protein metabolism and fermentation efficiency. These findings suggest that pea grains can be a sustainable and effective alternative to conventional protein and energy sources, promoting efficient rumen fermentation and nutrient utilization in dairy cows while potentially reducing reliance on imported feed ingredients.

## 1. Introduction

Pea (*Pisum sativum* L.) seeds have long been recognized as valuable feed ingredients for animal diets due to their high-quality protein and starch digestibility [1]. This legume is available in various forms, such as raw, split, ground, and extruded, among others, and offers relatively high concentrations of crude protein (CP: 17.9–24.1%) and well-balanced essential amino acids, along with significant energy content in the form of starch [1]. The protein content of these legumes is susceptible to rapid degradation within the rumen, thereby increasing the levels of rumen-undegraded protein (RUP) while preserving amino acid availability and resistant starch. Moreover, it can be hypothesized that the application of heat and pressure during the processing of these legumes could enhance their nutritional value for ruminants [2]. This legume could show interesting agronomic traits, such as high yield, uniform seed color and surface, as well as resistance to diseases and environmental stressors, making it adaptable to various growing conditions. These characteristics make pea grains an effective alternative to traditional protein and energy sources such as soybean meal (SBM), corn grain, or barley in dairy cow rations. Soybean meal (SBM) and corn grain are commonly used ingredients as concentrates for dairy cows in many parts of the world, providing high amounts of metabolizable energy and protein, becoming a key component of feeding programs. Nonetheless, despite the increasing specialization of livestock farms, feed costs remain significantly high. Consequently, it is necessary to find alternatives that can reduce feeding costs while meeting the animals’ nutritional requirements. Indeed, feed represents the most significant expense in milk production, particularly when taking into account the volatility of feed and milk markets [3]. In particular, during the previous year, soybean and its by-products, as well as cereal products, underwent several price fluctuations due to the geopolitical scenario [4]. Consequently, there is an imperative to explore alternative protein and energy sources for ruminant diets.

In dairy cows’ diets, pea grains can partially replace soybean meal and cereal grains without health risks or performance declines [5].

The use of alternative sources of plant protein rather than soybean meal in diets for livestock animals aims to reduce soybean imports into the EU and the carbon footprint of dairy cattle [4,5,6]. Thus, this study aimed to evaluate the partial replacement of soybean and corn meals with different varieties of pea grains in the diets of dairy cows.

## 2. Materials and Methods

### 2.1. Experimental Design

Three different pea (*Pisum sativum* L.) grain commercial varieties, namely Ganster, Peps, and Poseidon, were selected and analyzed through in vitro studies to assess their nutritional value. All three varieties have been selectively bred for advantageous agronomic traits such as high yield and good protein content. The Ganster, Poseidon, and Peps protein pea varieties were cultivated in the Campania region in Southern Italy, which is characterized by a Mediterranean climate with mild winters and hot, dry summers. This study was conducted in well-drained loamy soils, which are ideal for pea cultivation. Sowing took place in autumn, depending on the specific growing cycle, with moderate rainfall and possible supplementary irrigation when necessary. Fertilization included phosphorus and potassium, while natural nitrogen fixation was supported by symbiotic bacteria in the plants’ roots.

Each variety was ground (1 mm) and then included as flour into three distinct experimental diets (Table 1) for dairy cows formulated according to Sauvant and Nozière [7], partially replacing soybean meal and corn meal. These diets were compared with a control diet consisting of soybean meal and corn meal to evaluate their effectiveness. These experimental and control diets were isoproteic and isoenergetic.

### 2.2. Chemical Analysis

The three pea varieties (Table 2) and four diets (Table 3) were ground to pass through a 1.1 mm sieve and then analyzed. Dry matter (DM) content was determined at 103 °C (ID: 2001.12), along with crude protein (CP, ID: 978.04), ether extract (EE, ID: 920.39), starch (ID: 996.11), and ash (ID: 930.05), using AOAC [8] methodologies. Neutral detergent fiber (NDF) content was measured following the procedure described by Van Soest et al. [9]. Non-structural carbohydrates (NSCs) were calculated using the formula: 100 − (%NDF + %CP + %EE + %Ash). The forage unit value for milk production (UFL) was estimated [5].

### 2.3. In Vitro Gas Production Technique

Following the protocol indicated by Groot et al. [10], experimental and control diets were incubated (two runs) at 39 °C under anaerobic conditions for 120 h (six replications per diet, 6 × 4 = 24, in each run) in 120 mL bottles with a buffer medium (75 mL), a reducing agent (4 mL), and dairy rumen liquor (10 mL) as inoculum [11].

In each run, the rumen liquor was obtained from three multiparous Holstein dairy cows (second lactation) at a slaughterhouse, following EU legislation [12], on the day the trial started. All animal-related procedures were approved by the Ethical Animal Care and Use Committee of the University of Naples Federico II (Protocol 2019/0013729, dated 8 February 2019).

The collected rumen fluid was placed in a preheated thermos flask and transported within one hour to the Feed and Animal Production Analysis Laboratory of the University of Naples Federico II. There, samples were pooled, filtered through cheesecloth, and injected into sealed incubation bottles. Gas production was measured in PSI throughout the incubation period using a manual pressure transducer (Cole and Palmer Instrument Co., Vernon Hills, IL, USA) and recorded 22 times at intervals ranging from 2 to 24 h.

**Figure 1:**
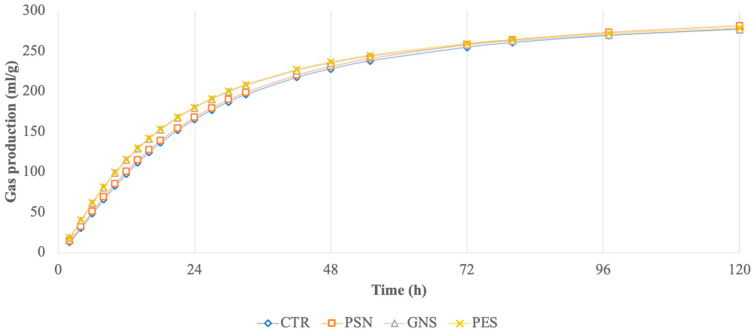
Cumulative in vitro gas production of control and experimental diets during 120 h of incubation.

Gas production shapes (Figure 1) were fitted to a sigmoidal model for each bottle stopped at 120 h, as indicated by Groot et al. [13]:

(1) G=A/(1+B/t)^C

where G is the total gas produced (mL per g of incubated OM) at time t (h), A is the asymptotic gas production (mL/g), B is the time at which one-half of A is reached (h), and C is the curve switch.

The cumulative volume of gas produced after incubation was expressed relative to the incubated organic matter (OMCV, mL/g iOM) and organic matter disappeared (yield, mL/g OMD). Organic matter disappeared (OMD, %) was determined by calculating the weight difference between the incubated organic matter and the undegraded filtered residue. Protein disappeared (CPD, %) was assessed as crude protein disappearance, measured using the Kjeldahl method [6].

**Figure 2:**
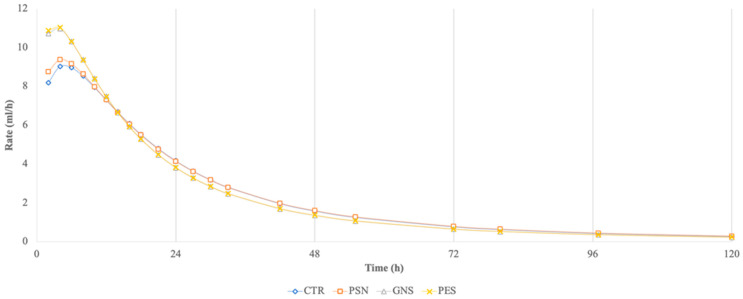
In vitro fermentation rate of control and experimental diets during 120 h of incubation.

The maximum fermentation rate (Rmax, mL/h, Figure 2) and the time at which it occurred (Tmax, h) were calculated utilizing model parameters [14]:

(2) $$Rmax=\frac{\left(A\times C^{B}\right)\times B\times {Tmax}^{\left(B-1\right)}}{\left(1+C^{B}\right)\times {\left(Tmax-B\right)}^{2}}$$

(3) Tmax=C×B−1B+11B

### 2.4. In Vitro End Products Fermentation

Fermentation liquor was collected from each bottle and cooled to 4 °C at the end of the trial. Subsequently, the liquor was centrifuged at 12,000× *g* for 10 min at the same temperature using a Universal 32R centrifuge (Hettich FurnTech Division DIY, Melle-Neuenkirchen, Germany) before analysis. The resulting supernatant was mixed with an equal volume of oxalic acid (0.06 mol) for volatile fatty acid (VFA) analysis. VFA concentrations were determined using a gas chromatograph (ThermoQuest 8000top, Italia SpA, Rodano, Milan, Italy) equipped with a fused silica capillary column (Supelco, 30 m, 0.25 mm ID, 0.25 μm film thickness). The external standard solution contained pure acetic, propionic, butyric, iso-butyric, valeric, and iso-valeric acids. The percentage of branched-chain fatty acids was calculated using the following formula: (iso-butyric acid + iso-valeric acid)/VFA × 100. Ammonia concentration was measured colorimetrically (Thermo Scientific, Helios Gamma 110–240 V, Waltham, MA, USA) at a wavelength of 623 nm, following the method described by Searle [15]. The reaction was initiated by adding phenol, followed by sodium hypochlorite as the oxidizing agent and sodium nitroprusside as a catalyst to enhance sensitivity. The pH was maintained between 10.5 and 11.5, and the reaction proceeded at 37 °C to accelerate color development. The intensity of the resulting blue indophenol dye was quantified spectrophotometrically, with calibration performed using ammonium standards to ensure precise measurement.

### 2.5. Statistical Analysis

The Shapiro–Wilk test was used to assess the normality of the data distribution. A general linear model (GLM) was performed, including each bottle as a random effect. Statistical analysis was performed using ANOVA to evaluate significant differences between diets. In cases in which significance was detected, Dunnett’s post-hoc test was applied to compare each experimental diet to the control diet, while Tukey’s test was used to compare the experimental diets with each other. All analyses were performed by JMP software (JMP, 2014).

## 3. Results

In Table 4, results obtained from 120 h of incubation are reported. GNS and PSE diets showed higher (*p* < 0.05) CPD than the CTR diet. All experimental diets had significantly (*p* < 0.05) decreased Tmax compared to the CTR diet (Figure 2). Similarly, PSG and PES diets were faster (*p* < 0.001) compared to the CTR diet. The PSN diet showed the highest (*p* < 0.05) value of Tmax and was the slowest (*p* < 0.001) of the experimental diets.

Table 5 shows the end products of fermentation. The experimental diets decreased (*p* < 0.05) ammonia production. The PSN diet showed the highest (*p* < 0.05) level of ammonia of the experimental diets. The PES diet increased (*p* < 0.001) VFA and acetate production compared to the CTR diet. Similarly, the PSN and PES diets had increased (*p* < 0.001) propionate production relative to the CTR diet. On the contrary, the GNS diet showed a lower (*p* < 0.05) level of VFA, acetate, and propionate compared to the control diet. The GNS and PES diets increased (*p* < 0.05) the butyrate level compared to the CTR diet. Comparing these experimental diets, the PES diet showed the highest (*p* < 0.001) levels of VFA, acetate, and propionate.

## 4. Discussion

As indicated by Zakoragis et al. [16], the cost and availability of imported soybean meal (SBM) and grain concentrates are heavily influenced by global trade dynamics, creating the need for more cost-effective alternatives. In this study, the inclusion of pea grains in the three experimental diets did not impact organic matter disappearance or in vitro gas production. However, the GNS and PES diets led to an increase in protein degradability after 120 h of incubation. This effect may be attributed to the high digestibility of peas in the rumen, which is comparable to that of soybean [6,17]. The protein content of these legumes is highly degradable in the rumen, leading to increased levels of rumen-undegraded protein while maintaining amino acid availability. Additionally, they can serve as an energy source, enhancing their overall nutritional value for ruminants [18,19]. Legume seeds, particularly pea grains, have the potential to provide readily available energy, which is especially beneficial for animals fed low-quality forages [20]. The experimental diets were found to improve fermentation kinetics, particularly the GNS and PES diets, which exhibited faster fermentation rates. Notably, the non-structural carbohydrates (NSCs) content in all diets (both control and experimental) exceeded 30% of dry matter (DM). Although the GNS and PSE diets contained less maize silage compared to the control diet, all diets were formulated to provide the same energy content. This indicates that the observed differences in fermentation kinetics were not simply due to the lower maize silage inclusion but were likely driven by the higher proportion of non-structural carbohydrates (NSCs), particularly starch, from pea varieties. The inclusion of these starch-rich ingredients can enhance microbial fermentation efficiency without compromising ruminal pH, ensuring that the energy supply remains balanced across diets. Therefore, the use of Ganster and Peps varieties allows for a reduction in maize silage without negatively affecting ruminal conditions or overall energy availability [21,22]. This acceleration is advantageous, as it promotes feed intake and nutrient absorption, ultimately improving growth performance and feed efficiency in ruminants. The faster fermentation kinetics observed in these pea-based diets may also indicate a more synchronized availability of nitrogen from protein and energy from NSCs, which is essential for maximizing microbial protein synthesis in the rumen [23]. Starch is classified into two main types: rumen-degradable starch (RDS) and rumen-escapable starch (RES) [24]. Pea grain is primarily composed of RDS [25], and an increase in RDS can influence both cellulolytic and amylolytic bacterial populations in the rumen, potentially elevating the risk of sub-acute ruminal acidosis [26]. However, the PSN and GNS diets did not affect ruminal pH levels, while the PES diet led to an increase in pH compared to the control diet, suggesting a low risk of acidosis.

Regarding fermentation end products, despite the increase in protein degradability, the experimental diets resulted in lower ammonia production compared to the control diet. As ammonia is the primary nitrogen source for microbial protein synthesis [27], this reduction may indicate improved utilization of NH_3_ by ruminal microbes, reflecting a balanced supply of energy and nitrogen [6]. This finding, particularly in relation to the GNS and PES diets, may be ascribed to the reduced alfalfa hay content inherent in these two diets. Indeed, even if the protein levels of these four diets are equivalent, the manner in which ingredients are combined could potentially enhance protein degradability in the rumen and reduce ammonia production.

Additionally, a shift in the microbial population composition was suggested by the increased production of volatile fatty acids (VFAs), particularly acetic and propionic acids, in the PES diet. Rumen fermentation primarily produces three VFAs (acetate, propionate, and butyrate) through the conversion of glucose derived from plant biomass [28]. This suggests that incorporating the Peps variety into the diet may enhance not only protein metabolism but also carbohydrate metabolism. Conversely, while the GNS diet led to a reduction in ammonia production, it also resulted in lower VFA concentrations, indicating a potential imbalance in the microbial population and a decline in fermentation efficiency. Nevertheless, all experimental diets increased butyrate production, which plays a crucial role in promoting rumen epithelial growth and function. Higher butyrate levels contribute to the development of larger rumen papillae and an expanded absorptive surface area, thereby improving VFA absorption [29].

## 5. Conclusions

Pea grains proved to be a viable alternative to soybean meal and cereal grain concentrates in dairy cow diets, maintaining organic matter digestibility and in vitro gas production. The GNS and PES diets increased protein degradability and improved fermentation kinetics, with no adverse effect on ruminal pH, reducing the risk of acidosis. All experimental diets reduced ammonia production, while the PES diet increased volatile fatty acid (VFA) production, supporting protein and carbohydrate metabolism. In addition, all experimental diets increased butyrate levels, promoting rumen health and nutrient absorption. When comparing these three experimental diets, Peps seems to be the most promising for improving protein metabolism and volatile fatty acid production. In conclusion, pea cultivars could be a good source of protein and an efficient source of energy, potentially reducing the need for additional energy-rich feed ingredients, which meet the nutritional requirements of dairy cow diets.

## Figures and Tables

**Table 1:** Percentage of ingredient inclusion in control and experimental diets (% fed basis).

	CTR	PSP	PSG	PSE
Maize silage	24.68	24.59	33.82	33.81
Alfalfa hay	54.64	54.45	44.94	44.93
Soybean meal	10.34	5.15	7.09	7.09
Corn meal	10.34	5.15	6.93	6.92
Pea grain var. Poseidon	0	10.66	0	0
Pea grain var. Ganster	0	0	7.22	0
Pea grain var. Peps	0	0	0	7.25

CTR: control diet; PSP: experimental diet with Poseidon variety; PSG: experimental diet with Ganster variety; PSE: experimental diet with Peps variety.

**Table 2:** Chemical composition of three varieties of pea grain.

Varieties	DM	CP	EE	NDF	Starch	Ash	NSCs
	%	% DM	% DM	% DM	% DM	% DM	% DM
Ganster	89.7	27.1	1.33	10.8	45.3	2.87	57.9
Peps	90.0	27.3	1.21	12.1	44.6	2.82	56.8
Poseidon	91.4	26.5	1.12	14.7	45.2	2.46	55.2

DM: dry matter; CP: crude protein; EE: ether extract; NDF: neutral detergent fiber; NSCs: non-structural carbohydrates.

**Table 3:** Chemical composition of control and experimental diets (% DM).

Diets	DM	CP	EE	NDF	Ash	UFL	NSCs
CTR	89.7	13.6	2.04	42.9	7.15	0.83	34.3
PSP	89.7	13.4	1.46	42.5	7.18	0.86	35.5
PSG	89.7	13.3	1.46	42.6	8.22	0.85	34.4
PSE	90.3	13.4	1.49	42.7	7.28	0.86	35.1

CTR: control diet; PSP: experimental diet with Poseidon variety; PSG: experimental diet with Ganster variety; PSE: experimental diet with Peps variety; DM: dry matter; CP: crude protein; EE: ether extract; NDF: neutral detergent fiber; UFL: milk forage unit; NSCs: non-structural carbohydrates (DM − (CP + EE + NDF + Ash)).

**Table 4:** In vitro parameters of tested diets after 120 h of incubation.

Diet	OMD	CPD	OMCV	Yield	Tmax	Rmax
	%	%	mL/g	mL/g	h	mL/h
CTR	72.4	81.6	261	362	4.69	9.13
PSN	72.0	83.8	265	359	3.97	9.62
GNS	72.1	85.1	262	356	3.15	11.1
PES	73.2	84.3	263	366	3.31	11.1
Dunnet test						
CTR vs.						
PSN	NS	NS	NS	NS	*	NS
GNS	NS	*	NS	NS	*	***
PES	NS	*	NS	NS	*	***
Tukey test						
	NS	NS	NS	NS	*	**
MSE	1.33	2.54	3.68	12.3	0.44	0.32

CTR: control diet; PSP: experimental diet with Poseidon variety; PSG: experimental diet with Ganster variety; PES: experimental diet with Peps variety; OMD: organic matter disappearance; CPD: crude protein degradability; OMCV: cumulative volume of gas related to incubated organic matter; Yield: cumulative gas production related to degraded organic matter; Tmax: time to reach maximum fermentation rate; Rmax: maximum fermentation rate. ***, **, and * indicate *p* < 0.001, *p* < 0.01, and *p* < 0.05, respectively; NS: not significant; MSE: mean square error.

**Table 5:** In vitro end products of tested diets after 120 h of incubation.

Diet	pH	N-NH_3_	VFA	BCFA	Ace	Prop	Iso-But	But	Iso-Val	Val
		mmol/L	mmol/giOM	mmol/giOM						
CTR	6.30	8.71	76.8	8.86	41.7	16.3	1.88	10.3	3.76	2.88
PSN	6.33	7.64	77.0	8.34	41.4	17.0	1.66	10.8	3.61	2.57
GNS	6.34	6.39	72.3	8.48	37.5	15.6	1.84	11.8	3.28	2.62
PES	6.36	6.77	83.2	7.98	45.4	17.9	1.81	11.7	3.62	2.75
Dunnet test										
CTR vs.										
PSN	NS	*	NS	NS	NS	**	NS	NS	NS	NS
GNS	NS	*	*	NS	*	*	NS	*	NS	NS
PES	***	*	***	NS	***	***	NS	*	NS	NS
Tukey test										
	NS	*	***	NS	***	***	NS	NS	NS	NS
MSE	0.17	0.29	1.19	0.78	0.67	0.15	0.06	0.56	0.47	0.12

CTR: control diet; PSP: experimental diet with Poseidon variety; PSG: experimental diet with Ganster variety; PES: experimental diet with Peps variety. iOM: incubated organic matter; N-NH_3_: ammonia; VFA: volatile fatty acid; BCFA: branched-chain fatty acids; Ace: acetate; Prop: propionate; Iso-But: iso-butyrate; But: butyrate, Iso-Val: iso-valerate acid; Val: valerate. ***, **, and * indicate *p* < 0.001, *p* < 0.01, and *p* < 0.05, respectively; NS: not significant; MSE: mean square error.

## Data Availability

Data are available on request.

## Abbreviations

The following abbreviations are used in this manuscript:

Ace	Acetate;
AOAC	Association of Official Analytical Chemists;
BCFA	Branched-chain fatty acids;
But	Butyrate;
CP	Crude protein;
CPD	Crude protein degradability;
CTR	Control diet;
DM	Dry matter;
EE	Ether extract;
GNS	Ganster;
Iso-But	Iso-butyrate;
Iso-Val	Iso-valerate;
MSE	Mean square error;
NDF	Neutral detergent fiber;
NS	Not significant;
NSCs	Non-structural carbohydrates;
OM	Organic matter;
OMCV	Cumulative volume of gas related to incubated organic matter;
OMD	Organic matter disappearance;
PES	Peps;
PNS	Poseidon;
Prop	Propionate;
RDS	Rumen-degradable starch;
Rmax	Maximum fermentation rate;
SBM	Soybean meal;
Tmax	Time to reach maximum fermentation rate;
UFL	Milk forage unit;
Val	Valerate;
VFA	Volatile fatty acid.
